# Elimination of HCV in Russia: Barriers and Perspective

**DOI:** 10.3390/v14040790

**Published:** 2022-04-11

**Authors:** Vasily Isakov, Dmitry Nikityuk

**Affiliations:** 1 Federal Research Centre of Nutrition, Biotechnology and Food Safety, Department of Gastroenterology & Hepatology, 109240 Moscow, Russia; dimitrynik@mail.ru; 2 I. M. Sechenov First Moscow State Medical University, 119435 Moscow, Russia

**Keywords:** HCV, Russia, elimination program

## Abstract

Hepatitis C virus (HCV) is highly prevalent in Russia, representing the largest pool of hepatitis C patients in Europe. Effective treatment regimens with direct-acting antivirals can achieve HCV cure in all patients; therefore, in 2016 the World Health Organization proposed eliminating hepatitis C as a public health threat by 2030. However, only a small number of countries are on track to meet the WHO’s hepatitis C elimination targets by 2030 due to many barriers in healthcare systems. This review focuses on a discussion about the epidemiology of HCV in Russia, the economic burden of HCV-related diseases, and treatment access with particular reference to the barriers for the elimination of HCV.

## 1. Introduction

Hepatitis C virus (HCV) infection is highly prevalent worldwide. It causes hepatitis that is slowly progressing to liver cirrhosis, which is associated with high mortality and is a significant economic burden as the only curative treatment at this stage of the disease is liver transplantation. However, during the last decade, highly effective direct-acting antivirals (DAAs) have been developed, and they allow to cure the HCV infection nearly in 100% of patients independently of the stage of liver disease, viral load, or HCV genotype. Thus, the elimination of the HCV infection has become possible and allows to prevent transmission, morbidity, and mortality. However, the elimination of HCV in every country is highly dependent on many factors, such as HCV prevalence, the economic burden of HCV-related diseases, public awareness of the disease, economic and human resources for launching the elimination program, and even a political will. Economically developed countries with low HCV prevalence and well-organized healthcare systems have better chances to achieve the elimination goals developed by the World Health Organization by 2030, but other countries, especially those with high prevalence, have fewer chances for the elimination even by 2050, and Russia is one of them.

## 2. Epidemiology of HCV Infection in Russia

**Figure 1:**
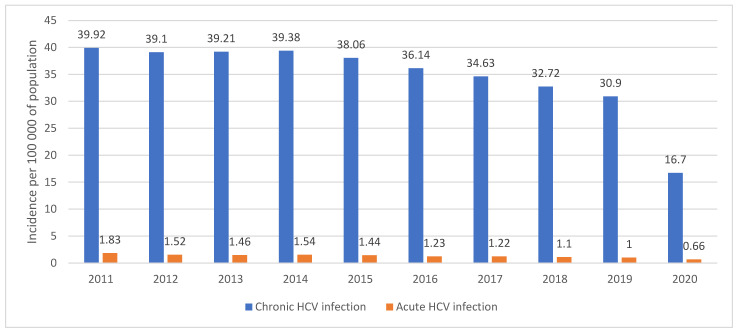
Incidence of chronic and acute HCV infection in Russia in 2011–2020 (per 100,000 of population) [6].

Russia is a large country with a population of more than 146 million, with a high HCV prevalence (4.1%) and the highest estimated number of HCV-infection cases in Europe (approximately 5 million) [1,2,3]. However, the real number of patients with hepatitis is unknown, although according to the official statistics report, 591,830 patients with chronic hepatitis C (CHC) were registered by the end of 2016 [4]. The incidence of HCV infection is high, but it has been gradually declining during the last 10 years. Thus, the incidence of acute hepatitis has decreased from 2.1/100,000 in 2010 to 0.66/100,000 in 2020 (Figure 1) [5,6]. High prevalence of HCV infection is typical for Eastern European countries, varying from 2% in Belarus to 5% in Ukraine [7]; however, due to the size of the population, Russia represents the largest pool of HCV patients in Europe. Globally, Russia, together with China, India, and Pakistan, falls into the top four countries with the highest number of HCV patients, including women of childbearing age [8].

In 2020, only 24,500 new CHC cases were registered, and the incidence rate was 16.7/100,000 [6], which is surprisingly low compared to the steady decrease in the incidence rate during the last ten years (Figure 1). It seems that the impact of SARS-CoV-2 on healthcare system functioning did not allow to reveal a typical number of new chronic HCV infection cases, which is likely explained by a decrease in the number of tests performed during the year, which was noted in many countries. In Georgia, the number of tests decreased by 51% [9], in Canada, HCV antibody testing (screening) and HCV RNA testing in 2020 decreased by 30% and 36% [10], and a huge reduction (>88%) in HCV tests was observed in the UK following the introduction of COVID-19 restrictions [11]. There were no specific studies published in Russia revealing the reasons for the decrease in HCV testing, however it could be explained by the several national lockdowns during the period we described above that led to restricted patients’ visits to GPs and reduced numbers of screening programs in the healthy population (which are the main source of new HCV antibodies test results). These lockdowns also changed infectious disease specialists’ practices, thus reducing the number of prescriptions for confirmatory PCR tests, because fewer patients were sent to them by GPs and there was an increase of competitive PCR testing for COVID-19. It is also important to note that PCR tests are reimbursed in Russia only when prescribed by specialists. Therefore, we think that the decreased number of HCV tests performed in this period provided the unexpectedly low incidence rate for 2020—the real incidence rate for 2020 should be very close to that of 2019, when 45,400 new cases were registered [5].

The incidence rate was highly variable between regions (less than 30 cases registered yearly in Ingushetia and more than 8000 in Moscow, for example). High geographical variability in the prevalence of HCV was confirmed by the study of 4764 blood samples from 5 Russian regions obtained from the healthy population. Anti-HCV antibodies were found in 2.6% (126/4764) of samples, and the prevalence of HCV varied from 1.3% to 3.3% in different regions; however, HCV-RNA was found only in 1.1% (50/4764) of samples, and there was no HCV-RNA found in children (0–14 years old), but there was an increase of HCV-RNA positivity in older age groups [12]. This long-term trend and a cohort phenomenon of a progressive annual decrease of CHC incidence was also found in a cohort of 20–29 years old, in which the incidence rate gradually decreased from 64/100,000 in 2011 to 38.3/100,000 in 2016 [4]. According to this analysis, nearly half of all CHC cases are registered in age groups from 30 to 49 years, which seems to be a decade younger than in Western European countries. 

The most prevalent HCV genotypes in Russia are genotype 1b (48.9–58%) and genotype 3a (34–39.6%), but genotype 2 (7.8%) and genotype 1a (3.7%) are rare [4,12]. HCV genotypes’ distribution is also influenced by the cohort phenomenon, whereby in older groups of patients HCV genotype 3a was found extremely rarely, but in the group of younger patients (30–39 years) nearly half were infected with the HCV 3a genotype [12]. Such distribution of genotypes among age groups might be partially explained by a different mode of transmission of HCV: the older population were primarily infected by blood transfusions, medical manipulations, etc., however, people who are now in their third/fourth decade of life were frequently infected due to intravenous drug use (IDU), which was an epidemic at the border of the century, when they were in their twenties. During the last 10 years, a substantial decrease in the use of opioids in favor of synthetics as well as the change of the intravenous route of taking illicit drugs to oral ones or to inhaling has been noted, especially in the young generation. This can also explain the decrease in HCV incidence in groups of people aged 20–29 years old and younger. According to recent state statistics, the prevalence of drug abusers was high (1293.35/100,000 in 2019), and 44.1% of them still used drugs intravenously [13]. Therefore, IDU is still one of the major modes of transmission of HCV in Russia. According to a modeling study, 100% of new HCV cases associated with IDU during 2018–2030 could be prevented if the additional HCV transmission risk attributable to IDU was removed [14]. This means that there should be a widespread syringe exchange program, opioid substitution program, and extensive treatment available for the risk groups in the population, with a special focus on people who injects drugs (PWID). However, in Russia, there are neither specific programs for the treatment of HCV in PWID, nor opioid substitution programs, and only 20 centers for syringe exchange exist in the whole country [14]. Unlike Russia, other Eastern European countries use opioid substitution programs, however, the extent of it is different from country to country. Thus, in Belarus, only 2 from 100 PWIDs are provided with opioid substitution therapy, which is lower than in Moldova or Ukraine. 

## 3. HCV Infection Consequences and Disease Economic Burden

The economic burden of HCV in Russia is supposed to be high, according to the estimated number of HCV-infection cases, prevalence of liver cirrhosis, and associated mortality. However, according to a State Report, chronic HCV infection holds only rank 13 among all infectious diseases, with an economic loss of 1.01 billion Russian rubles ($ 13.3 million) during 2020, which is much lower than for HIV, tuberculosis, and many other infectious diseases [6]. Even when the SARS-CoV-2 burden was not accounted for, such as in 2019, HCV infection held only rank 14, and therefore it has never been at the top of the list of the infectious diseases with a high economic burden in Russia (Supplementary Table S1).

Liver cirrhosis prevalence has increased dramatically in Russia, from 1521.2/100,000 in 1990 up to 2252.7/100,000 in 2017, with an estimated a total number of patients of 3,913,270 in 2017 according to systematic analysis [15]. It is quite difficult to evaluate the impact of HCV on this increase in liver cirrhosis prevalence and mortality in Russia, as there are no data available about HCV status in the statistical forms or death certificates of cirrhotic patients. The increase of mortality due to liver cirrhosis in many European countries, including Russia, was noted since the last quarter of the 20th century, and it was explained by an enormous increase of consumption of strong alcoholic beverages [16]. Combined mortality and alcohol consumption data analysis over a period of 35 years showed that changes in liver cirrhosis mortality in Russia in both sexes were highly dependent on the availability of vodka at the local market [17]. The most recent national publication indicated that the total number of registered patients with liver fibrosis and cirrhosis (except alcoholic) was 110,951 in 2015, with 18,640 new cases, and 34,673 patients died [4]. Obviously, in many of them liver fibrosis and cirrhosis were developed due to HCV infection, but it was far less than the number of cases which were projected by systematic analysis [15]. It seems that only in a minority (5% or even less) of patients was liver cirrhosis HCV infection the only and major cause of the disease development in Russia. For the development of a program of HCV elimination, it means that it is necessary to include the HCV status in all statistical forms, including death certificates of any cirrhotic patients, providing additional HCV testing to all cirrhotic patients who are registered, but still have not been tested, and providing immediate antiviral treatment to all HCV-positive patients. However, this approach will not dramatically change the total mortality rate due to liver cirrhosis in the nearest future, as in the majority of cirrhotic patients, alcohol consumption will be the main cause of the disease as well as its decompensation.

Liver cirrhosis is also prevalent in nearby Eastern European countries. According to a large systematic analysis, the prevalence of liver cirrhosis in 2017 in Belarus was high, 2031.3/100,000, and the estimated total number of patients was 231,686 [15]. Although the mortality due to liver cirrhosis in Belarus is the lowest among Eastern European countries, the same analysis showed that Belarus holds rank 3 (after Lithuania and Ukraine) among countries with the largest increases in the age-standardized death rate from cirrhosis over the period of 1990–2017. It was shown that at the end of the 20th century, cirrhosis mortality in Belarus was highly correlated with consumption and types of alcohol beverages, as in Russia, but later it was also influenced by additional factors and one of them was the epidemic of IDU, which resulted in the sharp increase of the incidence of HCV at the beginning of the 21st century and the later progression in some patients from chronic hepatitis to cirrhosis [18]. The analysis of the etiology of liver cirrhosis in one of the Belarus regions revealed that among cirrhotic patients with persistent disability, 35.5% of cirrhosis was developed due to viral hepatitis (91.8%—HCV, 1.8%—HBV, and 6.4%—mixed) [19]. The same study also noted the poor primary care diagnostics, as in 52.9% of cases liver cirrhosis was diagnosed due to decompensation. Among the patients with liver transplantation due to liver cirrhosis, the proportion of patients with HCV gradually increased during last 5 years of the study, up to 38% in 2016 [20].

Moldova demonstrated the highest estimated prevalence of liver cirrhosis in 2017 among Eastern European countries (3000.4/100,000), with an estimated number of patients with compensated liver cirrhosis equal to 137,489 [15]. The highest mortality due to liver cirrhosis among Eastern European countries was also noted in Moldova, where local studies indicated that it was even higher during the last 15 years, up to 98.5/100,000 [21]. This may be explained partly by the absence of a sex gap in the prevalence of liver cirrhosis as well as in the consumption of alcohol in Moldova in comparison to other countries. Liver cirrhosis alone accounts for half of the total life expectancy losses among adult Moldovan females. However, poor statistics on alcohol consumption, especially for homemade alcohol, and on viral hepatitis prevalence among cirrhotic patients make it difficult to evaluate the impact of HCV on cirrhosis genesis in Moldova.

There are no specific statistics of HCC in Russia, with the incidence and mortality evaluated according to the WHO ICD-10, in which malignant neoplasms of liver and intrahepatic bile ducts were united under the code C22. In the year of 2018, the incidence of tumors from class C22 was 3.31/100,000, with an average annual increase of 2.04%, and mortality was 3.66/100,000 [22]. It is possible that nearly half of these tumors were represented by HCC, however even the whole class of these tumors was not in the list of the top 7 malignancies according to their prevalence and mortality rate in the Russian population.

As in Russia, HCC is not highly prevalent in Belarus, it is neither in the list of the top 10 malignancies nor in the number of cases, nor for the mortality; however, it was mentioned that during the last 25 years, its prevalence increased by 13.9%, but it seems low in comparison to the increase in the prevalence of colorectal (159%) or prostatic cancer (620.9%) during the same period of time [23]. In 2016, HCC held rank 15 in men (1.2% of all registered malignancies in men) and 17 in women (0.7%), with a prevalence in both sexes of 5.1/100,000 [24]. There are no data about how many HCC cases were related to HCV.

Unlike Russia and other Eastern European countries, the highest primary liver cancer incidence both for men (12.5/100,000) and women (5.4/100,000) in Europe was registered in Moldova [25]. Besides the high prevalence of alcoholic liver cirrhosis, Moldova demonstrates higher prevalence for HBV and HDV in comparison to other European countries, which is obviously related to the high prevalence of liver cancer. A recent analysis of 139 HCC cases showed that 55.3% were HCV-positive, 36.1% were HBV-positive, and 18.5% were HDV-positive, but among all HCC cases in which any virus was detected, 25% of patients were co-infected [25]. Although 53.6% of patients in this study reported to consume alcohol, it seems that more than 80% of HCC cases were related to viral hepatitis.

## 4. Treatment of HCV Infection and Barriers to the Universal Access to the Treatment

All known DAAs combinations, except SOF/VEL/VOX, are approved in Russia, however the access to the treatment is restricted by the stage of liver disease (F3/F4) and financial resources of the region in which a patient lives. An infectious disease specialist or gastroenterologist/hepatologist usually prescribes treatment, and reimbursement is provided only in specialized centers based on the regional registry of hepatitis C patients. In total, in 2019, 6.2 billion rubles (USD 83 million) was spent on reimbursement for HCV treatment, which allowed to treat approximately 15,600 patients [26]. There were no DAAs generics approved in Russia before 2020, but under Russian law, individual citizens can import non-registered medicines for their personal use. Due to the restricted access to the reimbursement of HCV treatment, increasing numbers of Russians are treating their HCV infection with generic drugs produced in India, China, or Egypt, for which prices are ten times lower than the original drugs approved in the country. The efficacy of these generics is expected to be the same as in original drugs [27], however it is not possible to estimate the number of patients who treat themselves with generics as there are no statistical data. In 2020, local production and distribution of daclatasvir, sofosbuvir, and narlaprevir was started and it allowed to dramatically decrease the price tag for the SOF/DAC combination, which can be effectively used in non-cirrhotic patients with either HCV genotype 1 or 3. 

Major barriers for the successful elimination of HCV in Russia are the high prevalence/incidence of HCV, and the low treatment access. Low treatment access is due to unspecified healthcare policies and poor funding from local and central sources because decision makers do not recognize HCV infection as a big problem according to the low economic impact of HCV in comparison to other infectious diseases (HIV and tuberculosis, for example) or non-infectious diseases, which are at the top of the list of the most common causes of death in Russia. The major argument is that if half of the infected population are cured, it may produce a very small change in total morbidity and mortality, as the gap in numbers between the top 10 causes of disability and death and HCV-related diseases is huge in Russia. Still, there is no convincing analysis showing that expenses for the elimination program of HCV in Russia could produce the same or better results at the population level than the same funds being spent for screening of breast cancer or cardiovascular disease prophylaxis. However, the key for the change of the attitude of decision makers may be found in the change of prevalence of HCV in the future. If the situation with the treatment access is not changed, it is highly possible that the number of patients with HCV in Russia will have doubled by 2030. In order to prevent this unfavorable scenario, universal access to the treatment should be the first step, but it can be possible only if several pangenotypic DAAs generics are approved, and any doctor/nurse/pharmacist can prescribe it. Next, universal diagnosis of HCV in at-risk groups, such as IDUs, and providing treatment to all infected persons should be performed as soon as possible because it will break the chain of transmission of HCV and dramatically decrease the incidence, which can help to control infection in the future. 

In 2021, the national program for the elimination of HCV was launched with the plan to start its realization in 2023. This program includes changes in statistical forms to understand better where the HCV patients are in the healthcare setting, including HCV screening in specific subgroups related to increased transmission of the virus, organizing regional HCV patients’ registries, and increasing the treatment access through them, making it possible to treat 50,000–75,000 patients annually. It is also mentioned that the major DAAs combinations as generics or by license from the manufacturers should be produced locally. However, decentralization of treatment access is not clearly demonstrated in the program, which will be a possible bottleneck, whereby the patients will have the right to access the treatment but will have to wait a long time for the appointment with a specialist to obtain a prescription.

We think that the decentralization of testing and treatment access are the key factors in Russia for the success of the elimination program. The Russian Federation consists of 85 regions, which are extremely different by population density, economy, HCV prevalence, number of doctors and nurses per capita, and healthcare coverage, which leads to huge difference in access to the testing and treatment between regions. The same was noted in Canada, where healthcare is delivered regionally (provincially), and as such there are major policy differences across the country that affect the provision of HCV care. It was shown that both physicians and nurse practitioners can prescribe DAAs in 90% of provinces, however only 60% and 30%, respectively, are actively prescribing them. Paperwork requirements for treatment initiation continue to delay treatment and deter novice prescribers. In Canada, 80% of provinces require paperwork, and 40% require proof of investigations for treatment coverage [28]. Therefore, it is necessary to launch regional elimination programs in Russia to provide the best possible HCV cascade of care for every region, excluding any delay in testing and treatment reimbursement. 

Large studies on decentralization in different countries showed excellent results. In a Malaysian study of 15,366 adults screened in 25 primary care centers, significant differences in treatment initiation (71.0% versus 48.8%, *p* < 0.001) and treatment completion (90.5% versus 56.1%, *p* < 0.001) were found according to whether patients were treated at primary healthcare practices or at hospitals, respectively [29]. Treatment can also be prescribed by a pharmacist, such as in one Canadian province [28], or in the USA [30], which speeds up the HCV cascade of care.

Decentralization can also provide the HCV cascade of care in full in specific high-risk groups of patients, such as prisoners. A large study of 15,396 inmates who were screened at the 9 central prisons of Punjab (India) showed good outcomes across the care cascade, and decentralization of treatment in prison settings has improved the uptake and completion of treatment by the prison inmates [31]. During 2020, in the UK, High Intense Test and Treat (HITT) programs were performed at 9 prisons, with 4724 prisoners tested out of a potential 4858 (97%, range 96–100%). Among them, 422 (8.9%) were HCV-Ab-positive and 85 (20.1%) were viremic, and in 65 (76.5%) of them, treatment was started [32]. In 2020, more than 519,000 inmates were registered in Russia, with an estimated number of HCV-positive inmates of 40,000, as a minimum, representing a large source for the transmission of HCV. It is necessary to include this population group as a special part of the national HCV elimination program, providing the HCV cascade of care in full, as is already performed for TBC and HIV.

The planned increase of treatment access declared in the national program with the aim to treat at least 50,000–75,000 patients every year is not sufficient to overcome the negative trend, with 40,000+ new HCV cases registered annually. Besides the immediate treatment of patients at risk of disease progression (those with liver fibrosis F3–F4), the program should provide the HCV cascade of care in full to all PWID, as they are the main drivers of transmission and therefore a key for the reduction of HCV incidence. It can be provided not only through specialists in narcology, allowing them to prescribe DAAs, but also through civil organizations against illicit drugs use [33], by fast-track micro-elimination programs in drug rehabilitation centers [34], or through directly observed therapy, which combines DAA therapy with opioid agonist therapy to improve adherence to antiviral treatment [35]. All these approaches have confirmed their efficacy in different healthcare systems, and Russia should not be an exclusion.

In conclusion, Russia has a lot of barriers for the elimination of HCV infection, and the major one is the high prevalence of the infection. Additionally, the low access to the treatment in PWID and other groups of at-risk populations supports the high HCV incidence rate, and the unreasonable centralization of the healthcare system prevents from providing patients with the HCV cascade of care in full. The proposed national program should include not only an increase in the number of treatments provided to HCV patients, but also a decentralization policy for every region to speed up the cascade of care, involving any doctors and nurses, patients’ organizations, and volunteers. It should promote self-testing by rapid tests, knowledge, and education of society about HCV, using mass-media and social networks.

## Data Availability

Not applicable.

## Supplementary Materials

The following are available online at https://www.mdpi.com/article/10.3390/v14040790/s1, Table S1: Economic burden of infectious diseases in Russia in 2020. According to [6].
