# Supplementary material for: Elimination of HCV in Russia: Barriers and Perspective
Source: Viruses. 2022 Apr 11;14(4):790. doi: 10.3390/v14040790 (PMC9024583; doi:10.3390/v14040790)
Supplement: Supplementary file 1 [file viruses-14-00790-s001.zip › viruses-1592260-supplementary.pdf]

Table S1: Economic burden of infectious diseases in Russia in 2020. According to [6].

Rank	Disease or the Group of the Diseases	Economic Loss (Thousands of Rubles)
1.	Acute respiratory infections	606,505,442.0
2.	Tuberculosis (newly diagnosed cases), active	26,654,372.3
3.	Chickenpox	18,158,645.2
4.	HIV	12,091,161.8
5.	Acute intestinal infections, caused by unidentified pathogens	9,637,213.3
6.	Gastroenteritis caused by rotaviruses	4,271,046.6
7.	Animal bites	3,715,542.3
8.	Acute intestinal infections, caused by identified bacterial or viral pathogens	2,301,468.2
9.	Infectious mononucleosis	2,299,817.4
10.	Pediculosis	1,897,499.4
11.	Salmonellosis	1,593,376.7
12.	Influenza	1,577,740.0
13.	Chronic HCV infection	1,015,736.1
14.	Lyme disease	598,601.6
